# Tobacco control in the Russian Federation- a policy analysis

**DOI:** 10.1186/1471-2458-13-64

**Published:** 2013-01-23

**Authors:** Karsten Lunze, Luigi Migliorini

**Affiliations:** 1 School of Medicine, Boston University, 801 Massachusetts Ave Crosstown 2077, Boston, MA 02118, USA; 2 WHO Russian Federation, 9, Leontievsky pereulok, Moscow 125009, Russia

## Abstract

**Background:**

The Russian Federation (Russia) has one of the highest smoking rates in the world. The purpose of this study is to analyze past and current trends of the tobacco epidemic in the Russian Federation, review current tobacco control policy responses, and identify areas of opportunity for policy priorities.

**Methods:**

We used a policy triangle as analytical framework to examine content, context, and processes of Russian tobacco control policy. The analysis was based on secondary data on supply and demand sides of the Russian tobacco epidemic, tobacco-related economic and health effects during Russia’s economic transition, and compliance of Russian tobacco policy with international standards and regulations.

**Results:**

Tobacco-promoting strategies have specifically targeted women and youth. Russia’s approval of a “National Tobacco Control Concept” and draft for a comprehensive tobacco control bill increasingly align national legislature with the WHO Framework Convention on Tobacco Control (FCTC). However, several structural and cultural factors represent substantial barriers to the policy process. The influence of transnational tobacco companies on policy processes in Russia has so far impeded a full implementation of the FCTC mandates.

**Conclusions:**

Several strategies have been identified as having the potential to reduce the prevalence of tobacco use in Russia and decrease tobacco-related national health and economic burden: adjusting national tobacco policy by raising tobacco tax from the current lowest level in Europe to at least 70%; consequent enforcement of a complete smoking ban in public places; marketing restrictions; and smoking cessation interventions integrated into primary care. Russia’s tobacco control efforts need to target women and youths specifically to efficiently counter industry efforts.

## Background

Tobacco use is the single largest cause of preventable death globally, responsible for more than six million deaths each year, including more than 600,000 nonsmokers worldwide who die from secondhand exposure to tobacco smoke [1]. The Russian Federation (Russia) has one of the highest smoking rates in the world, particularly among men, with more than 39% or 44 million adults smoking in a country of 142 million [2]. 25% of Russian youth currently smoke [3].

These rates are higher than in any other European country. While tobacco use prevalence among males has been very high in the Russian Federation for the last 50 years, it has increased during the economic transition following the collapse of the Soviet Union in 1991. According to the Russia Longitudinal Monitoring Survey, tobacco smoking prevalence among males rose from 46-48% in the mid-1980s [4] to the current rate of over 60% [2].

**Figure 1:**
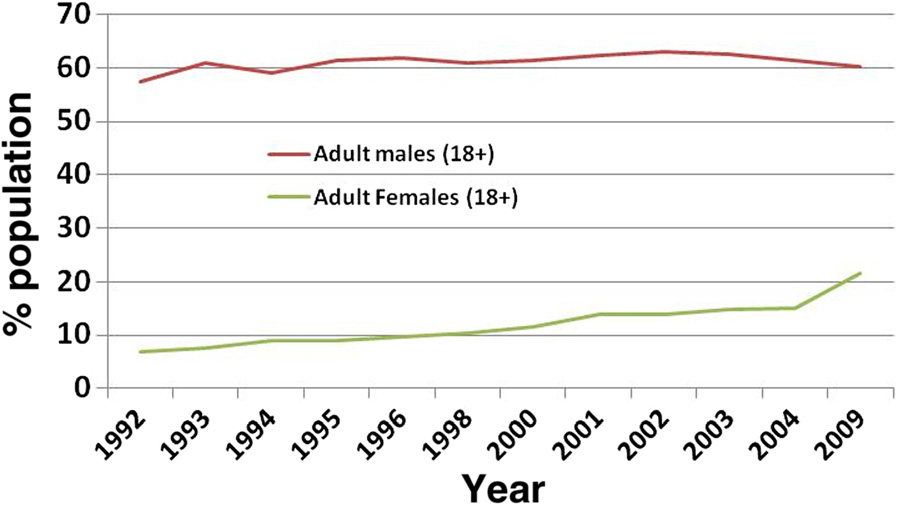
**Trends of adult smoking rates in Russia, 1992–2009.** Data from [2] and [5]. Label X axis: Year. Label Y Axis: Population.

Trends have increased even more among women, in whom rates before the transition had been historically low. Between 1992 and 2003, rates increased by 6% among men, but more than doubled from 6.9% to 15% among women (Figure 1) [2,5]. Since the 2000s, rates have been relatively stable among men, but further increased among females to the current rate of 21.7% [5].

Studies examining tobacco control policies in Europe have shown that understanding health policies requires analysis of its context, including political, economic, social and cultural influences, as policy contexts determine form and content of a policy and the attention it receives in the political arena [6]. Given the immense burden of smoking and tobacco use in Russia, the goal of this review is to conduct a policy analysis in order to provide an understanding of current tobacco control responses in Russia and identify areas of opportunity for effective tobacco control policies.

## Methods

We use the policy triangle analysis methodology [7] to examine Russia’s tobacco control policy content, context, actors and processes in a conceptual framework. Our analysis is based on publicly available, secondary data covering the timeframe from 1990 to present. As has been suggested previously [8], data include not only published academic sources, but also important policy documents and other reports from government and public institutions in Russia, from international organizations, as well as from transnational tobacco companies and Russian industry lobby organizations.

Drawing from peer-reviewed and grey literature from diverse areas that use a variety of research designs, and on the background of our experiences framed by existing theories and models [7-9], we provide a summary of supply and demand sides of the Russian tobacco epidemic, discuss tobacco-related health effects since the country’s economic transition, and analyze compliance of Russia’s tobacco policies with international standards and regulations.

### Policy context

#### *Situational factors*

**Tobacco demand** The vast majority of tobacco in Russia is consumed in the form of smoking, mostly of manufactured filtered cigarettes [2]. Less than 1% of consumed cigarettes are the previously common non-filtered cigarettes and papirosies, a local variant that uses a paper mouthpiece instead of a filter [10]. One of the reasons for the switch toward filtered cigarettes is the increasingly affordable retail price compared to other basic-needs goods. While income and real wages have been increasing by 12 to 16% annually, real prices for cigarettes have fallen by more than 40% over the last decade [10]. According to the most recent available price analysis, retail prices for packs of 20 cigarettes start at 4 rubles (RUB) (US$ 0.13 at a currency exchange rate of 30 RUB for 1 US$) for non-filtered cigarettes, and in 2010 averaged about $1.53 for a pack of the most sold brand [11]. Smokers on average spend more than RUB 560 rubles (US$ 18.70) per month on manufactured cigarettes, which would buy them subsidized commodities such as 37 loaves of bread, 16 liters of milk, or 2 kilograms of cheese or meat [2].

Since its economic transition after the end of the Soviet Union, Russia has ranked far ahead of all other countries in the Eastern European and Central Asian Region in per-capita cigarette consumption [12]. Initially, the decreasing domestic production of cheaper cigarettes in combination with an increasing demand for imported brand cigarettes created an incentive for illegally smuggled and counterfeit foreign tobacco products. At the same time, smuggling was also supply driven, as transnational tobacco companies used illicit supply to further penetrate the market [13]. Per capita consumption was about 1,550 taxed cigarettes in 1996. In 2000, taxed sales were reported at 1,700 cigarettes per capita, and total consumption including illicit products was estimated at up to 2100 sticks per capita, with 20-45% of all cigarettes available thought to be sold illegally [14].

**Figure 2:**
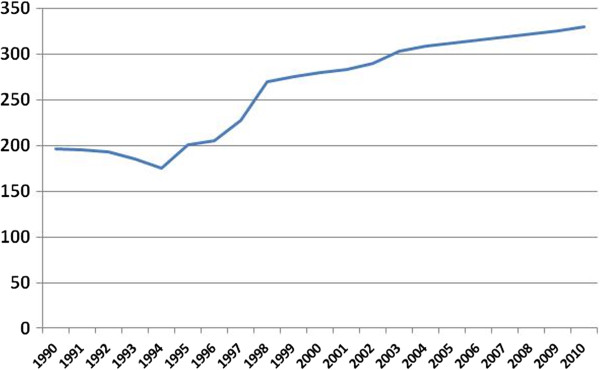
**Total cigarette consumption in Russia in billion sticks, 1990 – 2010.** Data from [2,5,10]. Label X axis: Year. Label Y Axis: Production in billion sticks.

Total cigarette consumption in Russia (Figure 2) has since increased further. Per capita consumption has reached more than 2200 cigarettes (or 125 packs) per year, and estimations of annual spending on tobacco products in 2005 range from RUB 83.4 billion (US$ 2.8 billion) [10] to about RUB 180 billion (US$ 6 billion) [15]. The opportunity costs of smoking, or money spent on the purchase of tobacco products that could be spent on other goods, amounted to about 0.9% of Russian GDP in 2009 [2]. Other costs add to the immediate economic loss from smoking. Wage data from Russia, adjusted for potential confounders, show that men who smoke earn about 14.8% less than non-smokers [15]. Lost productivity from tobacco-related premature death, estimated at RUB 710.4 billion (US$ 23.7 billion) per year, accounts for an additional 3% of GDP [10]. These numbers do not include the economic burden of tobacco-related morbidity, which presumably accounts for a substantial amount of the 5.4% of GDP spent on health by Russia [16].

**Tobacco supply** The opening of Russian markets to foreign investors during the political and economic transition from a socialistic planned economy towards free markets provided Western transnational tobacco companies the opportunity to enter an established market of male smokers. Former Soviet countries also offered a potential to expand the market by escalating the traditionally low female smoking rates [17]. Due to the breakdown in distribution networks and lack of spare parts for deteriorating production machinery, the domestic tobacco industry nearly collapsed [14], while the Russian economy defaulted on its debts in August 1998 and imports plummeted. Some propose that in the summer of 1990, the resulting cigarette shortage prompted smokers in major cities to take to the streets in what is now known as the Russian Tobacco Rebellion. Then-president Mikhail Gorbachev appealed to Western tobacco companies for “emergency” cigarette imports into Russia [18]. Others consider this account inflated and suggest that Boris Yeltsin, president after Gorbachev, later ordered almost all tobacco factories operating in Russia closed for renovation, which resulted in a purposive shortage of tobacco products and public outrage, and created an opportunity for foreign companies to appease the shortage through imports [19].

**Figure 3:**
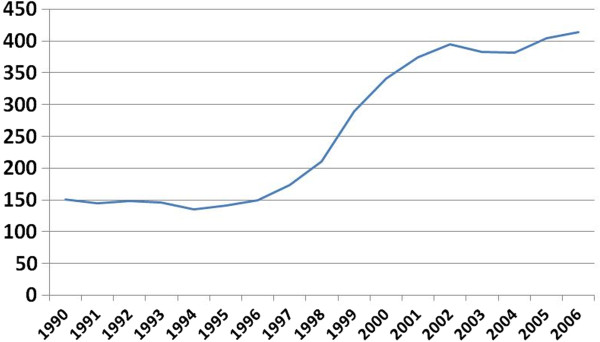
**Domestic cigarette production in Russia in billion sticks, 1990 – 2006.** Data from [2,5,10]. Label X axis: Year. Label Y Axis: Production in billion sticks.

Thus, transnational tobacco companies were among the first foreign investors in the Former Soviet Republics [20] and exponentially increased their imports, which soon reached 45% of total tobacco products sold in Russia. By the time the Russian tobacco state monopoly was dissolved at the end of 1993, domestic production by transnational tobacco companies was low and the market relied on imports (Figure 3). In subsequent years, transnational tobacco industry invested heavily, at least US$ 1.7 billion, in the Russian market [17]. With joint ventures between the local companies and foreign investors, the cigarette market transitioned from import-based to domestic production. The domestic industry almost tripled its output from 141.1 billion cigarettes in 1995 to 414 billion in 2006 (Figure 3). Investment by foreign companies largely facilitated production capacity.

In spite of Russia’s declining population, cigarette consumption increased overall by 81% during the economic transition between 1990 and 2000, surprising even transnational tobacco companies [17]. Companies have taken advantage of low import duties for raw tobacco and now produce 98.5% of all cigarettes domestically with imported tobacco leaves [10]. Currently, transnational companies (mainly Japan Tobacco International, Phillip Morris, British American Tobacco and Imperial Tobacco) control over 90% of the Russian tobacco market, while national companies such as Donskoy Tabak have recently increased their market share [21]. This increase might be linked to recruitment of markets in politically unstable regions such as South Ossetia and Abkhazia, where currently no tobacco control legislations are in place [22]. In the context of political instability, tobacco companies seize the opportunity of lack of tobacco control. Donskoy Tabak approached the Abkhaz parliament to engage in health-related projects, proposing to invest in a sanatorium there [23].

**Health effects** During the economic transition, Russia experienced unprecedented increases in mortality rates [24]. While life expectancy in Russian men has recovered from a low of 57 years during the transition to a pre-perestroika level of 64 years today [25], it still by far compares unfavorably to their male Western European counterparts with a life expectancy of up to 77 years [26]. Smoking has been suggested to be one of the most prominent factors explaining this East–west mortality divide [27]. Tobacco use ranks third in risk factors for total death and for total DALYs in Russia [26] and kills an estimated 332,000 people a year, mostly from cancers, respiratory, and cardiovascular diseases [28]. Smoking accounts for 30% of all male and 4% of all female deaths, shortening life expectancy by 6.7 years in men and 5.3 years in women [10].

The associated costs are immense: cardiovascular and respiratory diseases alone have incurred RUB 125 billion (US$ 4.2 billion) in costs to the Russian health system, while tobacco taxes have only amounted to RUB 20.3 billion (US$ 700 million) in proceeds [10]. Although recent trends signal some improvements in mortality rates and life expectancy [29], Russia’s unprecedented, tobacco-mediated health crisis affects primarily the middle-aged population thus potentially serving as a barrier to economic growth, in addition to negatively impacting the economic well-being of individuals and households [26].

### Gender-related, age-specific, and cultural factors

In Russia, the tobacco industry’s marketing efforts specifically target women and youths. Smoking among Russian women is predominantly prevalent in urban areas, although the gap between urban female smoking rates is closing as female smoking in rural areas seems to become increasingly socially accepted [2,10]. Tobacco-promoting marketing campaigns allude to ideas of independence, emancipation, and physical attraction and conform to Russian beauty ideals. Around 100 brands marketing specifically to women have been introduced to the Russian market [30]. Slim cigarette products appeal specifically to women, deceptively suggesting less harm with “light” or “low tar” labels. Consequently, about a third of Russian female smokers believe that “light” cigarettes are safer than regular cigarettes [2,17].

Among youths aged 15 to 18 years, smoking is more prevalent among boys than girls (30.1% vs. 17.8%) [2] and associated with alcohol use and maternal smoking in both sexes [31]. There are no tobacco-free public places in Russia, not even schools or health care facilities. More than a quarter of youths in Russia (27.5 %) are exposed to secondhand smoke at home and more than half of all adults are exposed to secondhand smoke in public places [2]. There are no data on mortality from secondhand smoke in Russia specifically. Extrapolating data from other countries, we estimate that secondhand smoke adds about 15% of mortality from active tobacco use to the burden of tobacco-related disease in Russia [10], translating to close to 50,000 additional deaths. Like women, youths are also specific targets of tobacco-promoting marketing campaigns, which liken cigarettes to lollipops and use other trivializing strategies [22].

### Policy process and policy actors

In the mid-1990s, several initiatives failed to develop tobacco prevention and control programs. At the time, the Federal Ministry of Health presented these program ideas as recommendations to the regional government, but did not allocate federal funding toward their implementation [32]. Starting in 1999, the State Duma (Russia’s Federal Parliament), and notably the then chairman of its Health Protection Committee, Nikolay Gerasimenko, tried to introduce a national tobacco control legislature that was initially drafted to resemble the FCTC draft. As a consequence of what was later called “a textbook demonstration of the lobbyist’s art” [33], the limitations on advertisements included in the initial bill and other proposed tobacco control measures were eventually removed, when the Federal Law No. 87-FZ of July 10, 2001 on the “Imposition of Restrictions on Tobacco Smoking” was passed in the Duma [34]. The tobacco industry remained an important influence in the policy process over the period that followed. Federal Law No. 38-FZ of March 13, 2006 “On Advertisement” provides key provisions governing advertising of tobacco products. Nonetheless, warnings on packs remained small and did not include graphics.

Several representatives of the Russian Federal Ministry of Health and the Federal Ministry of Foreign Affairs actively contributed to the development of the FCTC drafted by WHO. Remarkably, a British American Tobacco (BAT) employee was among the Russian delegation [35]. At the 56th Session of the World Health Assembly in May 2003, the Russian delegation voted for the adoption of the FCTC. In the period to follow, WHO in Russia facilitated high-level policy dialogue and provided technical support to the Russian Federal Ministry of Health and Social Development (MoHSD) and other national key counterparts in order to coordinate efforts of the Russian Federation towards joining FCTC. However, Russia initially did not sign the convention [35], which is the prerequisite for ratification. After years of campaigns by organizations such as the Russian Public Health Association, the Russian Anti-Tobacco Coalition, and the Russian Academy of Medical Sciences [36,37], Russia was one of the last of the currently 172 countries to sign the FCTC in 2008 [38].

Eight months later, the Duma passed a law drafted by tobacco industry employees [39,40]: Federal Law No. 268-FZ of December 22, 2008 “Technical Regulations for Tobacco Products”, providing definitions of key terms, packaging and labeling, compliance, and enforcement. The law conflicted with the FCTC to allow the misleading labels “light” and “mild”. Gennady Onishchenko, Chief Public Health Officer at the Ministry of Health, lamented this as a “shameful compromise between medical professionals and a criminal tobacco industry” [41].

The tobacco industry maintains a considerable influence in the State Duma, and many parliamentarians and senior members of the government support legislature favoring the tobacco industry. In fact, the director of the only remaining domestic tobacco manufacturing company in Russia (Donskoy Tobacco) was a member of the State Duma and co-authored all legal drafts concerning tobacco control [39]. The director of one of the most prominent tobacco industry lobby organizations recently became deputy of the Duma by taking over the mandate of a departing deputy of the leading party [40].

Russian non-governmental organizations have long called attention to the tobacco industry’s influences on law and policy making. Organizations such as the Russian Association of Public Health have tried to counteract the tobacco industry’s influence against stronger tobacco control [42] and demand greater transparency and disclosure of interactions between industry and public stakeholders [43]. An earlier bill proposed in the Duma defining alcohol and tobacco as illicit drugs and “genocide tools” did not find general support [32]. The Russian Anti-Tobacco Coalition recently brought a case before the Russian Supreme Court that tobacco is an unsafe and harmful product and its sales should thus be illegal. This claim was rejected, but has received some attention [39].

On 23 September 2010, Russia’s Prime Minister Putin approved the “Concept of the Government Policy on Combating Tobacco Use for 2010-2015”, which mandates the government and the Duma to pass legislation bringing Russia into full alignment with the FCTC and the FCTC Guidelines and specified an action plan to make Russia 100 percent smoke-free in indoor public and work places, and public transport; a complete ban on all forms of advertising, promotion and sponsorship for tobacco products; and mandated graphic health warnings on all tobacco packaging by 2015 [44]. The concept also formulated a 10-15% reduction in smoking by 2015; although not legally binding, this goal was considered “a strategic platform for future legislative steps” by the WHO [18]. In May 2012, the MoHSD submitted a tobacco legislation bill to the Central Office of the Government of the Russian Federation [45]. The bill is largely influenced by the “Concept” and closely aligns with various evidence-based policies for tobacco control proposed in the FCTC. After only two days, the bill was returned to the MoHSD and suspended based on “technical arguments” brought forth by the Ministries of Agriculture and Economic Development. In Russia, the hidden tobacco industry lobby targets these two ministries more heavily than it targets the MoHSD [18]. Against resistance from the tobacco industry [46], the MoHSD revised its tobacco control legislation draft, proposing an implementation of stepwise control measures during a transition period from 2014 through 2017 [45].

Kirill Danishevsky, the chairman of the Russian Anti-Tobacco Coalition which endorses the MoHSD’s proposal, related the suspension of the bill to the tobacco industry’s powerful resistance against public smoking bans and marketing restrictions through a number of non health-related ministries – such as the Ministry of Economic Development and Trade, the Ministry of Culture, and the Ministry of Agriculture [47]. The Economic Ministry, for example, has objected to the bill as causing financial losses for the national foreign trade, excessive administrative barriers and unwarranted costs for both entrepreneurs and the public budget [48].

The Head of the International Confederation of Consumer Societies, Dmitry Yanin, concludes that no effective anti-tobacco measures have been adopted in Russia over the past 20 years due to the influence of the tobacco lobby a on behalf of the tobacco industry to maintain a high volume of tobacco product sales. The Confederation also claims to have documents on the direct influence from the British American Tobacco Company on the position of the Russian Federal Ministries of Economy and of Industry and Trade on a number of clauses in the tobacco bill [47].

International NGOs such as the “Bloomberg Initiative To Reduce Tobacco Use” or the “Campaign for Tobacco-Free Kids” are supporting tobacco control in Russia, while organizations such as “Council on Issues of Development of the Tobacco Industry” or “Media Group Russian Tobacco” advocate on behalf of the tobacco industry.

### Policy content

#### *Monitoring*

The WHO established the Global Tobacco Surveillance System (GTSS) to assist country governments in surveillance and monitoring of tobacco control measures. Russia has strong technical capacity and appropriate implementing agencies to conduct surveillance. It was one of 11 countries to pilot test the Global Youth Tobacco Survey (GYTS) in 1999, which was repeated in 2004. Russia is also one of only 14 countries who recently conducted the first phase of the Global Adult Tobacco Survey (GATS) in 2009. The GATS and GTSS results provide internationally comparable data on tobacco control in Russia. The MoHSD has supported the idea of implementing the GTSS as part of the routine surveillance system for non-communicable diseases and according to the proposed bill, will allocate funds from the Federal Budget for the next phase of the GATS implementation [49].

#### *Exposure to secondhand tobacco smoke*

More than half of all Russians surveyed in the GATS reported that they had recently been exposed to secondhand smoke in public [2]. Currently, federal law in Russia prohibits smoking on the local metro and buses and restricts it in indoor workplaces, public places, and long-distance public transport, but lacks clear definitions of key terms such as “indoor,” “public place,” and "workplace". The Russian tobacco control draft bill mandates a complete public smoking ban, but allows for a transition period of 2–3 years for establishing ventilated designated areas for smokers. These, however, are known to incompletely protect nonsmokers [1].

#### *Tobacco cessation programs*

The GATS data estimate that more than 60 % of current Russian smokers are interested in quitting but only a third attempt [2]. Of those, only 11% successfully stop smoking, mostly on their own, only 20% with the help of pharmacotherapy and 4% with counseling [2].

With no smoking cessation support available in primary or inpatient care, there are few options for nicotine addiction treatment in Russia, where tobacco use like other drug addiction is still seen as a psychiatric disorder. As a consequence, tobacco use treatment is offered almost only at narcology facilities, or substance abuse clinics, where addiction psychiatrists use hypnosis, acupuncture and cognitive behavioral therapies to treat nicotine dependence. These clinics are primarily concerned with treating alcoholism and injection drug use, and offer limited medical treatment for smokers. Their services are not available in all regions and are largely considered ineffective and not evidence-based [2]. The existing private smoking treatment centers are prohibitive to most Russians because of their cost [50]. Russia is currently in the process of scaling up a national toll-free quit line, which already exists in St. Petersburg.

Nicotine addiction pharmacotherapies approved for the treatment of nicotine dependence include nicotine replacement therapy and varenicline, which are sold over the counter in any pharmacy store at a relatively high cost [1]. Another over-the-counter drug, available at a lower cost (less than US$8) is Cytizine, a nicotinic receptor agonist, marketed under the brand name Tabex and licensed in Russia for the treatment of tobacco dependence. Although it has been suggested to be effective for smoking cessation and have some potential for smoking cessation therapies in low- and mid-income countries due to its lower costs than other pharmacotherapies [51], the drug has remained largely unnoticed in the English-language literature and in countries outside of Eastern Europe and former Socialist states, where it has been used since the mid-1960ies [52].

#### *Tobacco labeling*

Cigarette package labels in Russia have been in compliance with the FCTC requirements text health warnings on the packaging to motivate smokers to quit. While current warnings do not include a picture or pictogram [53], in May 2012 the Russian Ministry of Health issued a decree (to go into effect in May 2013) mandating pictorial warnings, which will cover 50% of the back side of each pack [54].

#### *Advertising, promotion and sponsorship bans*

Russia’s tobacco industry invests massively in direct and indirect advertisements in various media, promotion, and sponsorship of events, estimated between US$ 60 million [10] and more than US$ 1 billion including all forms of product placement and sponsorships [19]. This is in spite of the FCTC mandate for a total tobacco marketing ban to protect youth from being drawn into tobacco consumption.

In Russia, federal law bans advertising for tobacco on television, radio and on outdoor billboards, and tobacco vending machines are generally prohibited. The new draft bill aims at closing current loopholes for tobacco companies to use billboards to advertise in the metro stations, in newspapers and magazines, and through other forms of marketing such as sponsorships of sports events, promotions, etc. This component is being contested by the tobacco lobby as harming the advertisement industry and reducing trade rather than tobacco consumption [48].

#### *Tobacco tax raises*

Based on vast research spearheaded by the World Bank, the FCTC stipulates that raising taxes and thus increasing tobacco product prices is the most powerful policy tool and most cost-effective intervention to reduce tobacco use [55,56]. As price elasticity is higher for people with low incomes, these strategies are particularly effective in preventing youth from starting, or convincing them to quit smoking [57]. Perhaps not surprisingly, increasing taxes on tobacco products in Russia is more supported by non-smokers (61%) than by current smokers (18%) [2].

**Figure 4:**
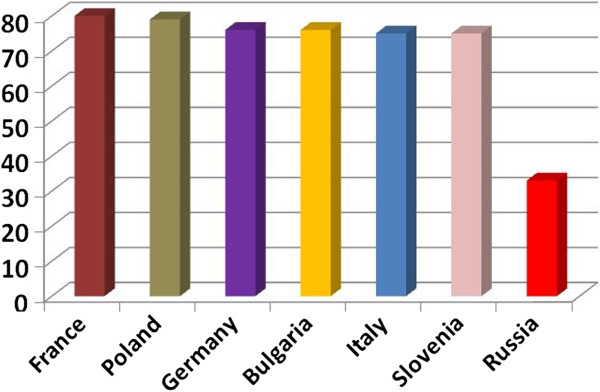
**Tax rates as percentage of retail price in various European countries in comparison to Russia.** Data from [10]. Label X axis: Country. Label Y Axis: Percent tax of retail price.

Cigarette taxation in Russia currently differs for filtered and non-filtered cigarettes and includes a specific excise tax (levied on a given quantity of tobacco, RUB 280 per 1,000 filtered and RUB 250 for non-filtered cigarettes), an ad valorem excise tax (based on a percentage of the retail price, 7% of the maximum retail price), and a value added tax (currently 18%), adding up to a total tax of 33 to 43% of the retail price for filtered and non-filtered cigarettes, respectively [10,11]. Although tobacco tax increased from 2010 to 2011 by 44% , from RUB 5 (US$ 0.17) per pack in 2010 to RUB7.20 (US$ 0.24) in 2011, these rates represent only about 10% of the rate in other European countries in the region (currently US $2.30) (Figure 4) and range far below the 67 to 80% of retail price recommended by the World Bank to efficiently reduce tobacco use [55].

The Bloomberg Initiative [58] and International Union Against Tuberculosis and Lung Disease [10], recommend a tax of 70% of retail price to maximize referring new onset of (youth) smoking and simultaneously revenue from taxes. However, the inflation-adjusted tobacco tax raise to 50% of retail price proposed in the draft bill, although closer to the European Region mean, lies below the recommended tax levels.

#### *Opportunities for tobacco legislation*

In Russia, as in many other countries, including mid-income countries [59], efforts from transnational tobacco companies have obviated evidence-based tobacco control legislation. Although like in other Eastern European countries, Russians’ knowledge of tobacco's deleterious health effects is limited, the public seems to largely support tobacco control measures [11]. A large majority of Russian adults (82.5%) in favors of a total advertisement ban [2]. The draft for a new tobacco control bill currently in preparation represents a formidable opportunity to effectively and efficiently reduce the prevalence of tobacco use among the Russian population, in order to counter commercial and other vested interests of the tobacco industry and decrease the tobacco-related national health and economic burden. Employing a modified multi-criteria decision analysis [60], we prioritize recommendations for future tobacco control policies and programs as ranked in Table 1 and discussed below. Given Russia’s leadership in the Eurasian Economic Community, policies should be harmonized within that organization.

**Table 1:** Policy priority ranking

**Intervention**	**Magnitude**	**Feasibility**	**Vulnerable populations**	**Evidence base**	**Costs**	**Score**
**Tax raises**	Total population	Low effort, resistance from industry	Women, children, youth		For enforcement	**12**
	+++	++	+++	+++	+	
**Smoking ban in public places**	Most of population	Difficult to enforce	Women, children, youth		For enforcement	**11**
	+++	+	+++	+++	+	
**Advertising ban**	Population amenable to marketing	Low effort, resistance from industry	Women, youth		Low	**10**
	++	++	++	+	+++	
**Warning labels**	Smokers amenable to risk communication	Low effort, resistance from industry	Women, youth		Low	**9**
	+	++	++	+	+++	
**Smoking cessation programs**	Smokers willing to quit	Need to train professionals	Effects from secondhand smoke		Program costs	**8**
	++	+	+	+++	+	

We developed the following criteria for prioritization of future tobacco control policies and programs: magnitude as estimated number of smokers and non-smokers affected; feasibility of policy change vis-a-vis expected political resistance or support from various stakeholders (such as parliament, ministries, administrations, scientific and professional organizations, non-governmental organizations, tobacco industry, etc.); expected impact on vulnerable populations such as youths or women; evidence base for effectiveness and cost-effectiveness; and projected costs associated with instating policies or implementing program in orders of magnitude.

The assigned scores, ranging from + (low effect, less desirable), ++ (medium effect), +++ (high effect, most desirable) for each equally weighed criterion, were summed up for each row to a total score.

### Policy opportunities for tobacco control in Russia

#### *Further increase in tax rates*

The inverse relationship between cigarette price and consumption is even stronger in Eastern Europe than in the West [61]. Raising the currently low (in comparison to other countries) tobacco tax to 70% of retail price represents an optimum in Russia to defer people from starting smoking, to prompt people to quit, and to increase tax revenues. If passed on to the consumer, this would increase the retail price by more than 120% [10]. By conservative estimates, tax revenues would increase by 300% and add RUB 153 billion (US$ 6 billion) in revenue annually, while potentially averting 2.7 million tobacco related deaths [10].

Empirical studies have shown that tax and subsequent price increases of 10% will reduce consumption by 8% in low- and middle-income countries [58]. Tax increases not only reduce consumption, particularly among poor and young people [62], they also increase government revenue, which can be earmarked for further tobacco control measures.

Tax increases will not lead to economic losses from decreases in demand and production, or to a net loss of jobs, but instead often generate new jobs and increase rather than decrease total revenues from taxes [55]. As evidence from other countries suggests, smuggling and other illegal sales will not negate these effects [63-66].

Price increases will have a disproportionate adverse economic effect on poor smokers who continue to smoke, but not all of them will be prompted to buy cheaper cigarettes notorious for higher nicotine and tar contents. Since individuals from lower socioeconomic strata are more responsive to price changes, more people with low income, particularly youths, will be discouraged from starting to smoke. The money not spent on tobacco will not be lost to the national economy. In a recent nationally representative survey, Russian smokers indicated that in the absence of smoking, they would spend available cash on groceries, recreation, housing and clothing [67].

#### *Smoking bans*

A complete smoking ban in public places consequently implemented and enforced, particularly in healthcare facilities and public buildings, with no exception for designated smoking areas, can effectively protect the public, particularly children and youths, from secondhand smoke. The currently proposed transition period of 2 to 3 years will have Russia lag behind the FCTC’s mandates for smoke-free environments [56], which have been found both effective and accepted in other European countries [68].

Recent nationally representative surveys of public attitudes towards tobacco control policies in Russia indicate that only a minority (14%) of respondents considered tobacco control adequate [17]. In contrast, more than half of all adults (59%) supported a total smoking ban in restaurants and almost half (49%) in bars [2]. With appropriate enforcement measures, compliance with smoking bans is usually high and people’s attitudes open towards policy change [68]. Other mid-income countries in Latin America found smoke-free policies to be cost-effective interventions to reduce both active and passive smoking [69].

#### *Advertisement bans and warning labels*

Russia’s intent to ban all tobacco advertisement and adopt its standard for graphic warnings on packaging to EU standards is consistent with attitudes of its constituents. Most Russians (87%), including smokers, perceive graphic warning labels on cigarette packages as highly effective and strongly support a government policy mandating these [70]. The vast majority of smokers (94%) noticed health warnings on cigarette package, however only a third (32%) indicated that the current text on warning labels made them contemplate quitting [2].

In addition, anti-tobacco counter-advertisements should publicize the detrimental potential of tobacco use. Social marketing campaigns in Russia targeted at cessation and second hand smoking have been shown to be effective, but were either temporary or pilot programs [50,71]. Recent campaigns have used public service announcement (PSA) materials from other countries to inform the public of the dangers of tobacco use and the options for and benefits of cessation. These could reach sizable audiences in Moscow and elsewhere in Russia at very limited costs [72].

#### *Integrating smoking cessation therapy into primary care*

Smoking cessation therapies in Russia are currently considered the responsibility of the narcology system, a remnant of the Soviet psychiatry system mainly concerned with the treatment of intravenous drug use and alcohol addiction. General health providers are not routinely trained in evidence-based smoking cessation interventions. Less than a third of smokers are advised by their health provider to quit [2]. Even specialist physicians lack training in smoking interventions and rarely offer cessation treatment [50]. Smoking cessation materials to support counseling strategies are often not available [50].

Physicians’ smoking habits fail to be a role model to Russians. In Moscow, more than 40% of all male and 13% of all female physicians smoke [73]. The Global Health Professional Survey (GHPS) conducted in the Russian Federation as part of the GTSS in 2006 showed that among third-year medical students (ages 19–20), 47% of males and 36% of females smoked [74]. In fact, Russian physicians often support the belief that “smoking is a free choice” and other messages that may serve as barriers to providing cessation counseling [17].

Tobacco use and nicotine dependency are increasingly understood as chronic addiction disorders. WHO recommends incorporating tobacco cessation services into primary health care, and to include behavioral counseling, nicotine replacement, low-cost or no-cost pharmacologic tobacco cessation therapies, and access to telephone quit help lines [56]. Materials to educate on brief smoking cessation interventions could be adapted from existing materials in other languages and integrated within the Russian primary health care and public health system. These efforts could be supported by readily available, toll-free telephone help quit lines that are already starting to be available in Russia.

#### *Monitoring & evaluation*

Given Russia’s strong capacity to appropriately monitor and evaluate the proposed policy changes, studies on the social and financial consequences of smoking should be conducted to inform decision makers on how to prioritize their policies.

## Conclusions

Russia’s “National Tobacco Control Concept” and a recent MoHSD draft for a new tobacco control bill represent a formidable opportunity to effectively and efficiently reduce the country’s tobacco burden. Our results suggest that strong tobacco industry influences risk to attenuate future tobacco control measures.

In order to implement the mandates of the FCTC and its “National Tobacco Control Concept” and to adopt its promising bill draft, Russia needs to strengthen national leadership for tobacco control through clear, evidence-based health messages on behalf of state and non-state actors. Although Russia has no domestic raw tobacco producers who will be deprived of their livelihood, it represents a global center of attention and interests from transnational tobacco companies; substantial policy resistance will originate primarily from cigarette producers and related marketing and distribution industries. Leaders can relate to the recent “National Tobacco Control Concept” to seek sustained political commitment and build strong coalitions to advance tobacco control in the country, with particular attention to tobacco use among women and youth.

Aligning the various stakeholders and harmonizing their collaboration has the potential to gain momentum beyond current policy resistance and industry influences. A successful tobacco control policy change with its positive changes can demonstrate Russia’s capacity to improve public health and address the current health crisis. Robust research is needed to create a solid evidence-base on the effectiveness of tobacco control measures in Russia.

## Competing interests

KL has no conflict of interest to report. He has not received any fees for conducting this analysis. LM is employee of the World Health Organizations, whose declared goal is to control the global tobacco epidemic.

## Authors’ contributions

KL and LM conceived of the study. KL conducted the analysis and drafted the manuscript. LM revised it critically and provided important intellectual content. Both authors read and approved the final manuscript.

## Pre-publication history

The pre-publication history for this paper can be accessed here:

http://www.biomedcentral.com/1471-2458/13/64/prepub
